# Sodium Caseinate Induces Apoptosis in Cytarabine-Resistant AML by Modulating SIRT1 and Chemoresistance Genes, Alone or in Combination with Cytarabine or Daunorubicin

**DOI:** 10.3390/ijms26157468

**Published:** 2025-08-01

**Authors:** Daniel Romero-Trejo, Itzen Aguiñiga-Sánchez, Amanda Velasco-García, Katia Michell Rodríguez-Terán, Fabian Flores-Borja, Isabel Soto-Cruz, Martha Legorreta-Herrera, Víctor Manuel Macías-Zaragoza, Ernesto Romero-López, Benny Weiss-Steider, Karen Miranda-Duarte, Claudia Itzel Sandoval-Franco, Edelmiro Santiago-Osorio

**Affiliations:** 1 Hematopoiesis and Leukemia Laboratory, Research Unit on Cell Differentiation and Cancer, Faculty of High Studies Zaragoza, National Autonomous University of Mexico, Mexico City 09230, Mexico; danielromerot19@gmail.com (D.R.-T.); liberitzen@comunidad.unam.mx (I.A.-S.); amanda.velasco.2000@gmail.com (A.V.-G.); katia.rodriguez.0199@gmail.com (K.M.R.-T.); iram66671@yahoo.es (V.M.M.-Z.); dr.ernestoromerolopez@gmail.com (E.R.-L.); bennyweiss@hotmail.com (B.W.-S.); karen_duarte15@hotmail.com (K.M.-D.); clau.ixel@icloud.com (C.I.S.-F.); 2 Department of Biomedical Sciences, School of Medicine, Faculty of High Studies Zaragoza, National Autonomous University of Mexico, Mexico City 56410, Mexico; 3 Centre for Oral Immunobiology and Regenerative Medicine, Barts & The London School of Medicine and Dentistry, Queen Mary University of London, London E1 2AT, UK; f.flores-borja@qmul.ac.uk; 4 Molecular Oncology Laboratory, Cell Differentiation and Cancer Research Unit, FES Zaragoza, National University of Mexico, Mexico City 09230, Mexico; sotocruz@unam.mx; 5 Molecular Immunology Laboratory, Computational Chemistry Research Unit, Faculty of High Studies Zaragoza, National Autonomous University of Mexico, Mexico City 09230, Mexico; marthal@unam.mx

**Keywords:** acute myeloid leukemia, chemoresistance, sodium caseinate, cytarabine, combined therapy

## Abstract

Resistance to cytarabine (Ara-C) remains a major obstacle to the successful treatment of acute myeloid leukemia (AML). Therefore, modulating Ara-C resistance is indispensable for improving clinical outcomes. We previously demonstrated that sodium caseinate (SC), a salt derived from casein, the principal milk protein, inhibits proliferation and modulates the expression of Ara-C resistance-related genes in chemoresistant cells. However, it remains unclear whether the combination of SC with antineoplastic agents enhances apoptosis, modulates chemoresistance-related genes, and prolongs the survival of tumor-bearing mice implanted with chemoresistant cells. Here, we investigated the effects of SC in combination with Ara-C or daunorubicin (DNR) on cell proliferation, apoptosis, the expression of chemoresistance-associated genes, and the survival of tumor-bearing mice. Crystal violet assays, quantitative reverse transcription polymerase chain reaction (qRT-PCR), immunofluorescence, flow cytometry, and Kaplan–Meier survival curves were used to evaluate the effects of combinations in chemoresistant cells. We demonstrate that the IC_25_ concentration of SC, when combined with antileukemic agents, increases the sensitivity of chemoresistant WEHI-CR50 cells to Ara-C by downregulating SIRT1 and MDR1, upregulating the expression of ENT1 and dCK, enhancing apoptosis, and prolonging the survival of WEHI-CR50 tumor-bearing mice. Our data suggest that SC in combination with antileukemic agents could be an effective adjuvant for Ara-C-resistant AML.

## 1. Introduction

Acute myeloid leukemia (AML) is a highly heterogeneous and aggressive hematological malignancy that originates from myeloid progenitor cells and is characterized by abnormal proliferation of undifferentiated cells [1]. Although cytarabine (Ara-C) and anthracyclines are key chemotherapy agents for AML treatment [2], the development of multidrug resistance and the adverse side effects associated with chemotherapy are major obstacles to the successful treatment of AML. Therefore, overcoming resistance to Ara-C is essential for improving clinical outcomes in patients [3].

Although the mechanisms underlying chemoresistance in AML are not yet fully understood, accumulating evidence indicates that reduced activity or loss of function of the equilibrative nucleoside transporter 1 (ENT1) and deoxycytidine kinase (dCK) contributes significantly to Ara-C resistance in both AML patients and leukemia cell models [3,4,5,6,7]. Additionally, SIRT1 (silent information regulator 1 homolog 1), an epigenetic regulator from the sirtuin family, play a crucial role in development of chemoresistance in AML, which enhances the overexpression of multidrug-resistance-associated transporters, including MDR1 and MRP1, making cancer cells resistant to the conventional chemotherapy [8,9,10,11]. Therefore, there is an urgent need to identify natural products capable of reversing chemoresistance in AML while minimizing toxicity to healthy tissues.

Casein, the major protein conforming milk, is a tumor suppressor capable of inducing apoptosis and decreasing proliferation, invasion, migration, and metastasis in several cancer types, including melanoma, breast, colorectal, ovarian, and AML [12,13,14,15]. We have demonstrated that sodium caseinate (SC), a salt derived from casein, inhibits the proliferation of several leukemic cell lines [16,17,18,19]. Moreover, SC also promotes in vitro proliferation of bone marrow mononuclear cells (BMMNCs) from BALB/c mice, demonstrating inhibitory effects on the proliferation of leukemia cells without damaging healthy cells [18]. Aguiñiga et al. [17] demonstrated that SC in combination with daunorubicin (DNR) has a synergistic effect, inhibiting proliferation in parental AML cells and improving the survival of tumor-bearing mice. However, it remains unknown whether any of these combinations have the potential to sensitize Ara-C-resistant leukemic cells. In this study, we analyzed in vitro the effect of combined therapy with SC, Ara-C, or DNR and explored the underlying mechanism of its therapeutic effect on cytarabine-resistant cells. Our findings demonstrate that the combined administration of SC with antineoplastic agents inhibits proliferation and enhances apoptosis in cytarabine-resistant WEHI-CR50 cells by decreasing the expression of genes involved in chemoresistance (SIRT1 and MDR1), increasing the expression of ENT1 and dCK, molecules involved in the uptake and metabolism of Ara-C, and increasing the survival of WEHI-CR50 tumor-bearing mice.

## 2. Results

### 2.1. Sodium Caseinate, Cytarabine, or Daunorubicin Inhibit the Proliferation of Chemoresistant WEHI-CR50 Cells

**Figure 1:**
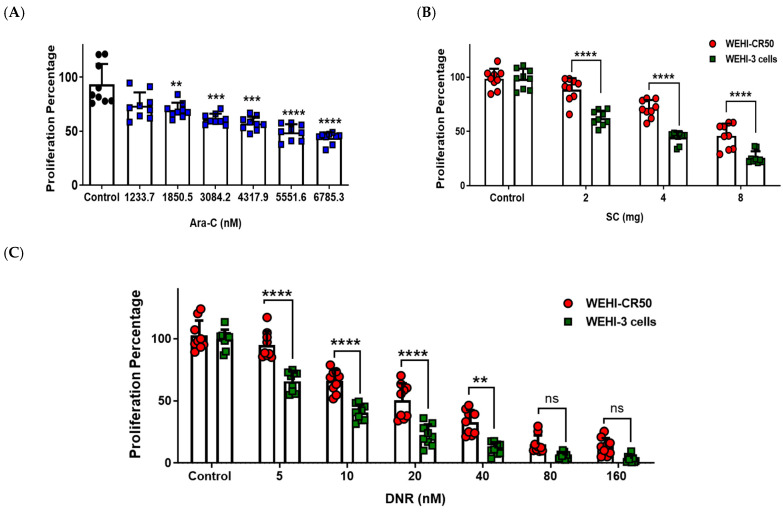
Proliferation percentage of parental WEHI-3 and chemoresistant WEHI-CR50 cells after treatment with Ara-C, SC, or DNR. (**A**–**C**) Cells were treated with increasing concentrations of Ara-C, SC, or DNR for 72 h, and the cell proliferation was assessed by violet crystal assay. Bars are means ± SD of individual data points from triplicates of three independent experiments. Statistical significance was assessed using one-way ANOVA followed by Tukey’s or Student’s *t*-test, ** *p* < 0.01, *** *p* < 0.001, **** *p* < 0.0001. ns, non-significant. Abbreviations: Ara-C, cytarabine; DNR, daunorubicin; SC, sodium caseinate; nM, nanomolar; mg, milligram.

As a first approach to evaluate the effects of SC, Ara-C, or DNR on the proliferation of both parental WEHI-3 and cytarabine-resistant WEHI-CR50 cells, we performed a cell proliferation assay. We observed a dose-dependent cytotoxic effect and a cross-resistance to SC and DNR in chemoresistant WEHI-CR50, which exhibited a significantly higher proliferation rate compared to the parental cells with the same concentration (Figure 1B,C). These findings suggest that WEHI-CR50 cells are a good model for evaluating the efficacy of SC against chemoresistant AML.

Statistical analysis of cytotoxicity data from chemoresistant WEHI-CR50 cells indicated that the IC_50_ values are 5420.5 ± 540 nM for Ara-C, 7.16 ± 0.69 mg for SC, and 16.7 ± 0.69 nM for DNR. The corresponding IC_25_ values are 1649.5 ± 152.9 nM for Ara-C, 3.59 ± 0.32 mg for SC, and 7.7 ± 0.58 nM for DNR.

### 2.2. The Combined Administration of Sodium Caseinate with Cytarabine or Daunorubicin Is More Effective Compared to Monotherapy in Chemoresistant WEHI-CR50 Cells

**Figure 2:**
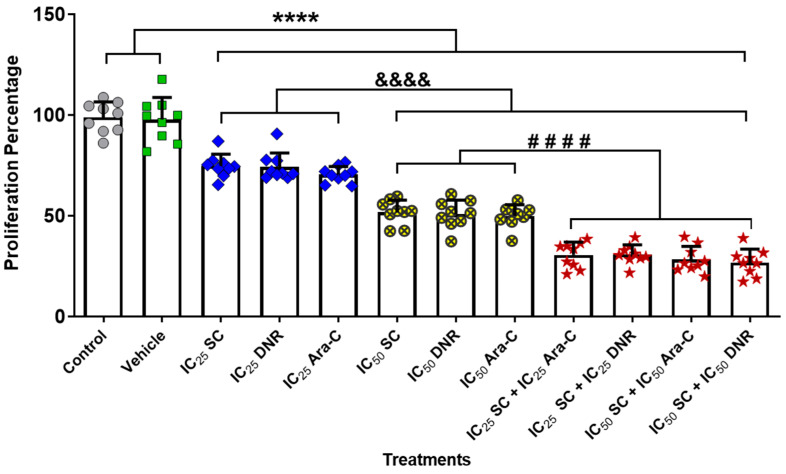
Proliferation percentage of chemoresistant WEHI-CR50 cells treated with IC_50_ and IC_25_ values of Ara-C, DNR, and SC, either alone or in combination. Cells were treated for 72 h, and the proliferation percentage was measured by violet crystal assay. Bars represent means ± SD of individual data points from triplicates of three independent experiments. Statistical analysis was performed using two-way ANOVA followed by Tukey post hoc test. **** *p* < 0.0001 indicates a significant decrease compared to the control group. &&&& *p* < 0.0001 indicates a significant decrease compared to the IC25 combinations. #### *p* < 0.0001 indicates a significant decrease compared to the IC50 combinations.

Since the combination of anticancer agents enhances cytotoxic effects compared to monotherapy in cancer cells [20,21,22,23,24,25], we evaluated the cell proliferation following combined treatment with SC + Ara-C or SC + DNR using the previously determined IC_25_ and IC_50_ concentrations in the chemoresistant WEHI-CR50 cells. We demonstrated that both monotherapy and combined therapy at IC_25_ and IC_50_ concentrations effectively inhibited the proliferation of WEHI-CR50 cells. Moreover, all combined treatments significantly reduced the percentage of cell proliferation by more than 60% compared to monotherapy (Figure 2). Statistical analysis using the GraphPad Prism 8 software revealed that the combined treatments of SC + Ara-C and SC + DNR at IC_25_ concentrations did not show a significant difference compared to the combined therapy at IC_50_ concentrations (Figure 1A–C). These results suggest that equivalent reductions in cell proliferation could be achieved using lower doses of chemotherapeutic agents when combined with the natural compound SC, potentially allowing for dose reduction of conventional chemotherapy. Given this promising finding, we opted to focus further analysis on the IC_25_ combinations for subsequent experiments.

### 2.3. Sodium Caseinate, Alone or in Combination with Antineoplastic Agents, Enhances Apoptosis in Chemoresistant Cells by Downregulating SIRT1 Expression

**Figure 3:**
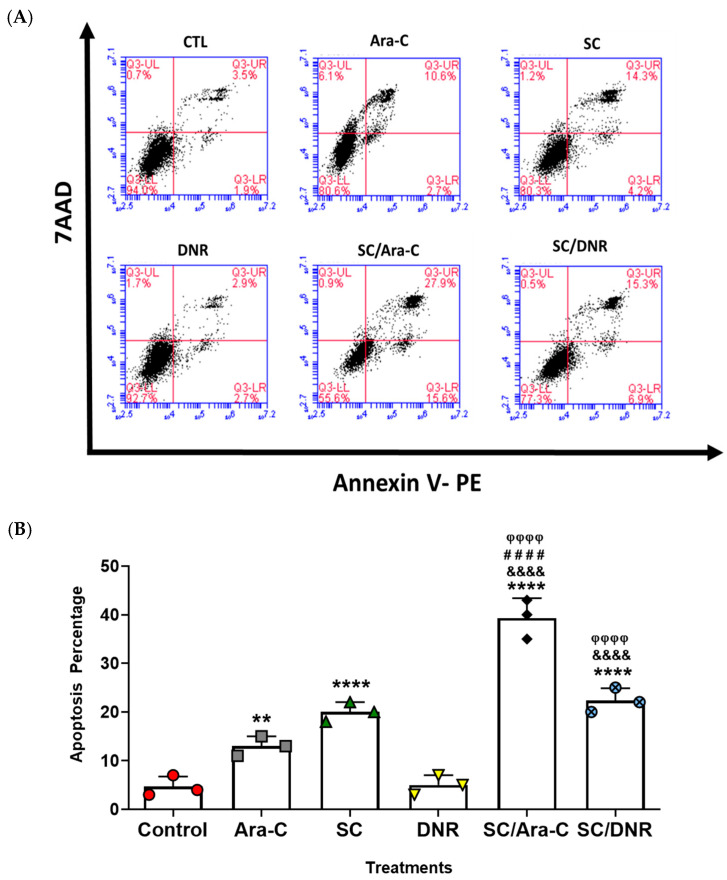
SC alone or in combination with Ara-C or DNR increases apoptosis in WEHI-CR50 cells. (**A**) Cells were treated with IC25 concentrations of Ara-C, SC, and DNR, either alone or in combination for 48 h, and apoptosis was assessed by flow cytometry. Representative dot plots show 7-AAD vs. annexin V. (**B**) Percentage of WEHI-CR50 cells in the presence of the treatments, either alone or in combination. Data are representative of three independent experiments, and values are expressed as mean ± SD. Statistical analysis was performed using two-way ANOVA followed by Tukey’s post hoc test. ** *p* < 0.01, **** *p* < 0.0001 vs. control group; &&&& *p* < 0.0001 vs. Ara-C group; #### *p* < 0.0001 vs. SC group; φφφφ *p* < 0.0001 vs. DNR group.

To test the effect of SC in combination with antileukemic agents on apoptosis induction in chemoresistant WEHI-CR50 cells, we performed flow cytometry analysis using the annexin V technique. We demonstrated that individual treatments at the IC_25_ concentration significantly induced cell apoptosis in 13% and 20% of the population treated with Ara-C and SC, respectively, while no significant change was observed with DNR treatment compared to the control group. However, a significant increase in the percentage of apoptotic cells was observed when the cells were treated with the IC_25_ combinations of SC + Ara-C or SC + DNR, inducing apoptosis in over 40% and 23% of the population, respectively, compared to the control group (Figure 3A,B). These data indicate that SC, either alone or in combination with Ara-C or DNR, reverses chemoresistance in WEHI-CR50 cells by increasing the percentage of apoptotic cells.

**Figure 4:**
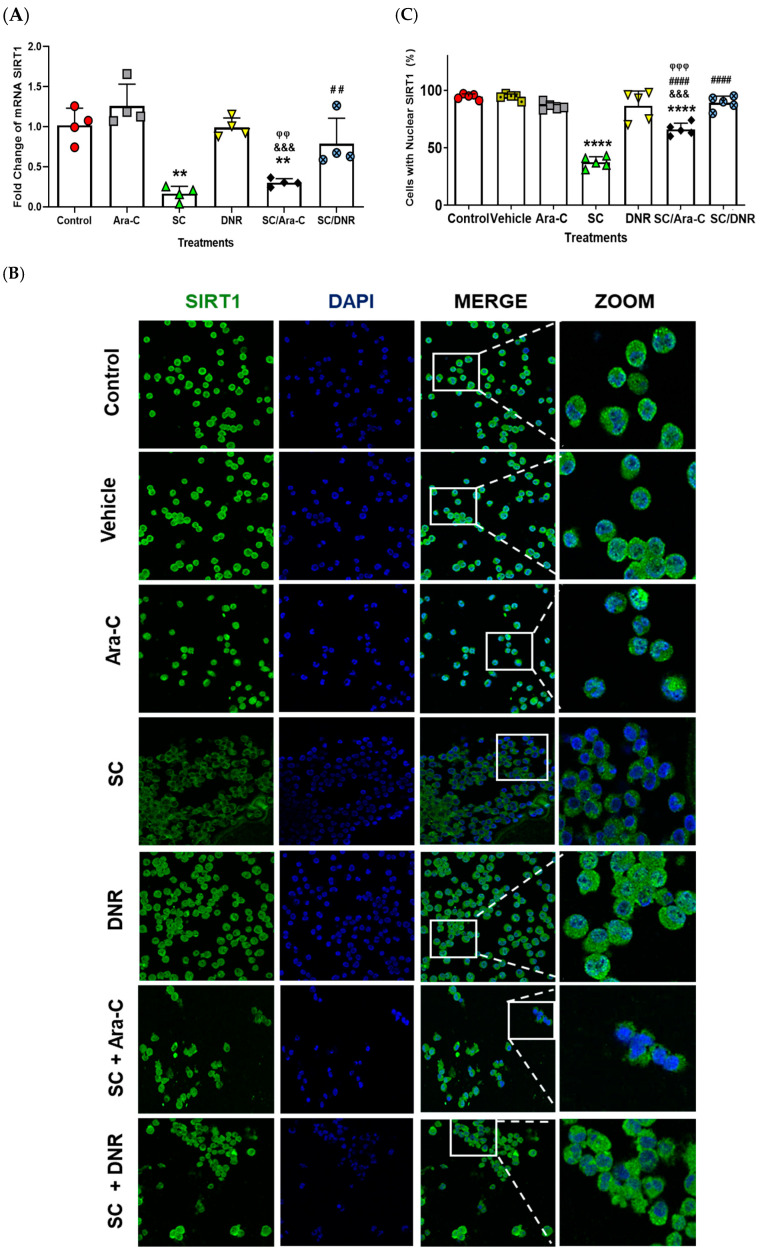
SC, alone or in combination with Ara-C, reduces the nuclear localization of SIRT1 in chemoresistant WEHI-CR50 cells. (**A**) Real-time RT-PCR analysis of sirt1 mRNA expression in WEHI-CR50 cells treated with IC25 concentrations of Ara-C, SC, or DNR, alone or in combination. (**B**) Representative images of confocal microscopy for SIRT1 (**left panel**); nuclei stained with DAPI (**middle panel**); and merged images (**right panel**). Scale bar: 50 µm. (**C**) Quantification of SIRT1 nuclear translocation was performed using the mean of the data of three fields per experimental condition. Data are representative of four independent experiments, and values are expressed as mean ± SD. Statistical analysis was performed using two-way ANOVA followed by Tukey’s post hoc test. ** *p* < 0.01, **** *p* < 0.0001 vs. control group; &&& *p* < 0.001 vs. Ara-C group; ## *p* < 0.01, #### *p* < 0.0001 vs. SC group; φφ *p* < 0.01, φφφ *p* < 0.001 vs. DNR group.

Since SIRT1 is an oncogene involved in regulating chemoresistance and proliferation in AML cells [9,26,27,28,29], we demonstrated using quantitative real-time reverse transcriptase–polymerase chain reaction (qRT-PCR) and immunofluorescence assays that SC alone or in combination with Ara-C significantly decreased SIRT1 expression compared to other experimental groups (Figure 4A). Moreover, we quantified the number of cells exhibiting nuclear SIRT1 localization using immunofluorescence techniques (Figure 4B,C). We observed that SC, either alone or in combination with Ara-C, significantly reduced nuclear SIRT1 localization compared to the other experimental groups (Figure 4B). These results suggest that SC, either alone or in combination with Ara-C, decreases the expression and nuclear translocation of SIRT1, attenuates cell proliferation (Figure 2), and enhances apoptosis in chemoresistant cells (Figure 3).

### 2.4. Sodium Caseinate in Combination with Cytarabine Reverse Chemoresistance by Increasing the Levels of ENT1 and dCK While Decreasing the Expression of MDR1

**Figure 5:**
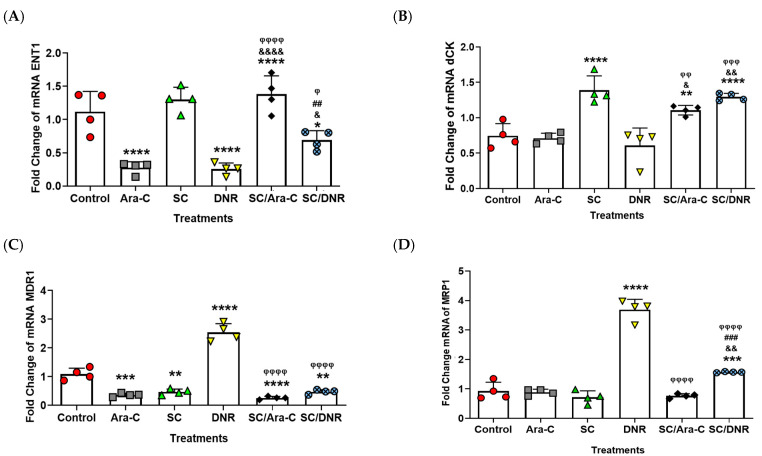
Combined treatments with SC and Ara-C or SC and DNR modulate the expression of genes associated with chemoresistance in WEHI-CR50 cells. (**A**–**D**) Real-time RT-PCR analysis of ENT1, dCK, MDR1, and MRP1 mRNA expression in WEHI-CR50 cells treated with IC25 concentrations of Ara-C, SC, and DNR, alone or in combination. Data are expressed as the fold change in relative mRNA expression from four independent experiments. Β-actin was used as a housekeeping gene for the quantification and normalization of each studied gene using the 2^−ΔΔCT^ method. Statistical analysis was performed using two-way ANOVA followed by Tukey’s post hoc test. * *p* < 0.05 ** *p* < 0.01, *** *p* < 0.001, **** *p* < 0.0001 vs. control group; & *p* < 0.05, && *p* < 0.01, &&&& *p* < 0.0001 vs. Ara-C group; ## *p* < 0.01, ### *p* < 0.001 vs. SC group; φ *p* < 0.05, φφ *p* < 0.01, φφφ *p* < 0.00, φφφφ *p* < 0.0001 vs. DNR group.

**Figure 6:**
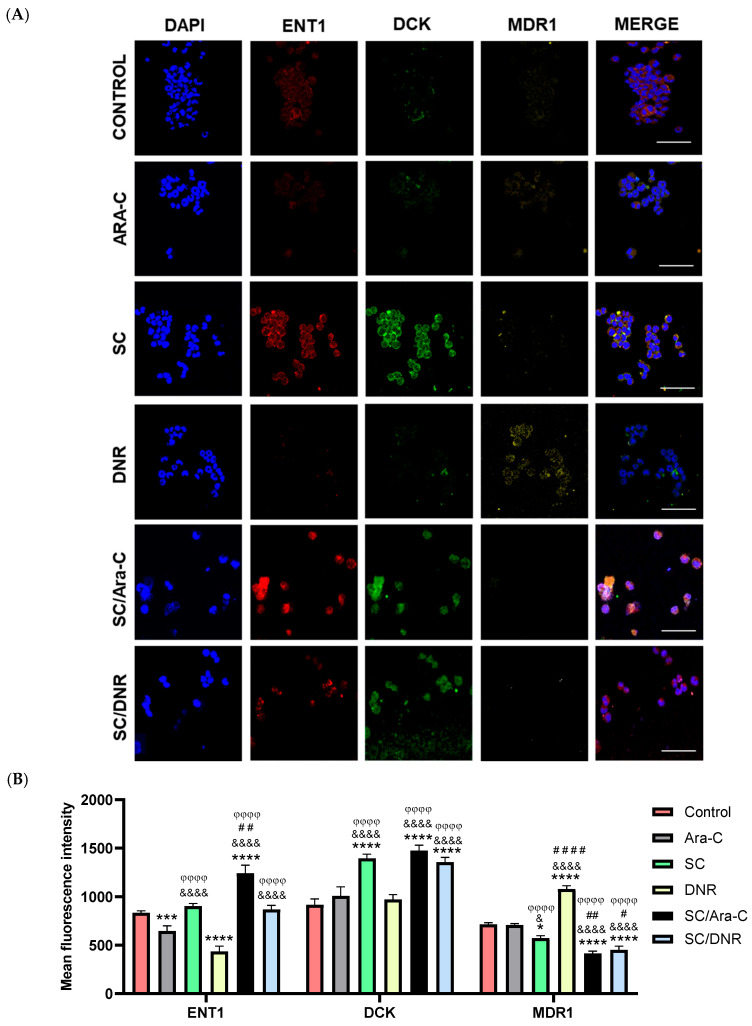
SC alone and in combination with Ara-C or DNR modulates the expression of ENT1, DCK, and MDR1 in chemoresistant WEHI-CR50 cells. (**A**) Representative confocal microscopy images showing the expression patterns of ENT1, DCK, and MDR1 in treated and untreated WEHI-CR50 cells. Merged images are shown in the right panel. Scale bar 50 µm. (**B**) Quantification of the proteins ENT1, DCK, and MRP1 was performed by measuring the mean fluorescence intensity from three fields per experimental condition. Data are representative of three independent experiments and are expressed as mean ± SD. Statistical analysis was performed using two-way ANOVA followed by Tukey’s post hoc test. * *p* < 0.05, *** *p* < 0.001, **** *p* < 0.0001 vs. control group; & *p* < 0.05, &&&& *p* < 0.0001 vs. Ara-C group; # *p* < 0.05, ## *p* < 0.01, #### *p* < 0.0001 vs. SC group; φφφφ *p* < 0.0001 vs. DNR group.

It is well-known in the literature that the overexpression of SIRT1 regulates the expression of several genes involved in chemoresistance [9,26,29]. To further explore this relationship and evaluate the impact of SC, alone or in combination with antileukemic agents, on chemoresistant cells, we examined the expression levels of equilibrative nucleoside transporter 1 (ENT1) and deoxycytidine kinase (dCK), two genes known to sensitize AML cells to Ara-C [3,30,31]. Furthermore, we assessed the expression of MDR1 and MRP1, two well-characterized efflux transporters that contribute to chemoresistance by reducing the intracellular accumulation of daunorubicin in cancer cells [32,33]. Based on both qRT-PCR and immunofluorescence assays, we found that combined treatment with SC and Ara-C significantly increased ENT1 expression compared to Ara-C or DNR monotherapy (Figure 5A and Figure 6). In contrast, SC alone did not significantly alter ENT1 expression relative to the SC + Ara-C group. Notably, individual treatments with Ara-C or DNR significantly reduced ENT1 expression compared to the combination treatments. Regarding dCK expression, treatment with SC alone or in combination with Ara-C or DNR significantly increased its expression compared to the other experimental groups (Figure 5B and Figure 6). In the case of MDR1, treatment with SC, SC + Ara-C, or SC + DNR significantly reduced its expression compared to the control or DNR groups. DNR monotherapy significantly increased MDR1 expression; however, the SC + DNR combination significantly reduced MDR1 levels relative to both the control and DNR-treated groups (Figure 5C and Figure 6). No significant differences in MRP1 expression were observed among most treatment groups compared to the control, with the exception of the DNR group, which showed a significant increase. Importantly, SC + Ara-C and SC + DNR treatments significantly decreased MRP1 expression relative to the DNR group (Figure 5D and Figure 6). These results indicate that SC, particularly in combination with Ara-C or DNR, sensitizes WEHI-CR50 cells to Ara-C by modulating key genes involved in chemoresistance.

### 2.5. Sodium Caseinate Combined with Antileukemic Agents Improves Survival in Tumor-Bearing Mice

**Figure 7:**
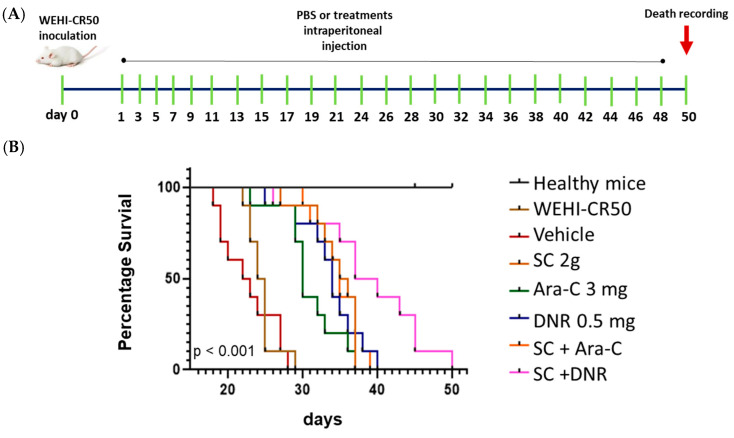
SC in combination with antineoplastic agents prolongs survival in WEHICR50 tumor-bearing mice. (**A**) Therapeutic experimental design to assess survival. Balb/C mice were inoculated intraperitoneally (i.p.) with 2.5 × 10^5^ WEHI-CR50 cells. A total of 24 h after cancer cell inoculation, animals were divided into several groups, and the different treatments were initiated and administered i.p. every 2 days for a total of 24 doses. Mice were monitored daily for survival, and the time of death (in days) was recorded. (**B**) Kaplan–Meier survival curves. The survival percentage of tumor-bearing mice injected with resistant WEHI-CR50 cells, followed by intraperitoneal administration of the different treatments, was evaluated. Statistical analysis was performed using the log-rank test. *p* < 0.001.

To assess the therapeutic potential of the combined therapy in vivo, we evaluated the survival of mice injected with chemoresistant cells. First, we injected WEHI-CR50 cells intraperitoneally in mice. A total of 24 h later, mice were administered intraperitoneally every 48 h for a total of twenty-four doses using the individual or combined treatments (Figure 7A). We followed the protocol of implantation and treatment previously described [17]. As shown in Figure 7B, the median survival time for tumor-bearing mice without treatment and those treated with PBS was 25 and 23 days, respectively. Treatment with either Ara-C or DNR alone extended median survival to no more than 34 days. Notably, mice treated with SC alone or in combination with Ara-C showed an increased median survival of 36 days, while a combination of SC + DNR resulted in a significant improvement, with a median survival time of 39 days. These results suggest that SC combined with DNR prolongs the survival of tumor-bearing mice.

We also want to highlight that treatment with SC, either alone or in combination with antineoplastic agents, not only inhibits the proliferation of cytarabine-resistant cells by enhancing apoptosis induction and modulating the expression of chemoresistance-related genes but also significantly improves the survival of WEHI-CR50 tumor-bearing mice. More than 20% of the treated animals survived to day 50, compared to survival limited to day 40 in the other groups, indicating a clear improvement in disease progression (Figure 7B).

## 3. Discussion

The multidrug resistance (MDR) phenotype remains a major challenge in cancer therapy, contributing significantly to treatment failure, disease relapse, progression, and metastasis in different cancer types [34,35]. In AML, despite the availability of conventional therapeutic strategies—including the 7+3 regimen, FLAG (fludarabine, cytarabine, and filgrastim), gemtuzumab ozogamicin (GO), allogeneic hematopoietic stem cell transplantation (allo-HSCT), and the liposomal formulation CPX-351—the disease often remains incurable [36,37,38]. These limitations underscore the urgent need for novel treatment strategies with improved efficacy, lower toxicity, and the ability to overcome chemoresistance. In this context, we previously demonstrated that sodium caseinate (SC) can modulate the expression of genes associated with Ara-C resistance in WEHI-CR50 cells [16]. However, it remained unclear whether SC, in combination with Ara-C or DNR, could effectively reduce the proliferation of chemoresistant cells, enhance apoptosis, and improve survival outcomes in vivo.

In this study, we observed that individual treatments inhibited cell proliferation in a dose-dependent manner in both parental and chemoresistant AML cells. Notably, the concentrations of SC and DNR required to achieve this effect were significantly higher in cytarabine-resistant cells, suggesting the presence of cross-resistance to multiple chemotherapeutic agents. Our observations are consistent with those reported by Illangeswaran et al. [39], who reported that cytarabine-resistant cells exhibit reduced sensitivity not only to Ara-C but also to DNR, Midostaurin, and arsenic trioxide. Similarly, HL-60 cells resistant to cytarabine showed cross-resistance to gemcitabine and cladribine [40], while MV4-11 cells exhibited resistance to both FLT3 inhibitors and a four-fold increase in resistance to DNR [39]. Gertjan et al. [41] also demonstrated significant cross-resistance between Ara-C and DNR in primary AML patient samples. These findings have important clinical implications, as they indicate that resistance to a single agent may compromise the effectiveness of other drugs, contributing to the development of multidrug resistance [42,43,44,45]. Therefore, strategies that reverse Ara-C resistance are essential to improving treatment outcomes in AML.

To the best of our knowledge, this is the first report demonstrating that SC, when combined with Ara-C or DNR, can sensitize chemoresistant AML cells. This effect was evidenced by reduced cell proliferation, increased apoptosis, and prolonged survival in tumor-bearing mice. Our findings align with previous reports showing that combination therapies targeting multiple cellular pathways simultaneously are more effective than monotherapies in overcoming resistance [42,46,47]. Interestingly, the combinations tested at IC_25_ concentrations exhibited synergistic antiproliferative effects comparable to those observed at IC_50_. No significant differences in proliferation rates were observed between the IC_25_ and IC_50_ combinations, suggesting that effective therapeutic responses can be achieved with lower doses of chemotherapeutic agents when combined with SC. These results are consistent with those of Aguiñiga et al. [17], who demonstrated that combination SC with antineoplastic agents at IC_25_ values produces synergistic effects comparable to those observed in parental WEHI-3 cells. This dose-sparing effect could be clinically advantageous, potentially allowing for a reduction in chemotherapy-related toxicity by minimizing off-target effects and preserving healthy cells. Supporting this hypothesis, we previously demonstrated that SC not only inhibits AML cell proliferation in the bone marrow but also promotes the recovery of healthy hematopoietic cells 30 days after leukemic cell inoculation [48]. Moreover, SC has shown immunomodulatory properties, notably stimulating macrophages and enhancing the production of TNF-α, a cytokine involved in tumor cell apoptosis and immune surveillance [49]. In earlier in vivo studies, SC treatment also significantly reduced hepatomegaly, splenomegaly, and solid tumor formation while increasing survival in mice inoculated with AML cells [18,19]. These findings highlight the potential of SC as a dual-function agent: a chemosensitizer capable of overcoming resistance mechanisms, and a hematopoietic modulator that supports immune and bone marrow recovery. This dual action may be particularly beneficial in the treatment of chemoresistant forms of leukemia.

On the other hand, understanding the molecular mechanisms underlying cancer progression and drug resistance is crucial for identifying key targets which could significantly impact drug resistance [50,51]. In this context, the overexpression of Sirtuin 1 (SIRT1), a NAD^+^-dependent deacetylase, has been shown to play an oncogenic role in various cancer types [9,28,32], including AML [8,26]. This oncogenic activity is mediated through the deacetylation of several transcription factors such as NF-κB, p53, FoxO1, AP-1, and JNK/c-Jun [8,26,29,32,52], which are known to negatively regulate the expression of drug resistance-associated genes including MRP1, MDR1, AKT, and BCL-2 [33,34]. The upregulation of these genes contributes to chemoresistance by enhancing survival pathways and drug efflux mechanisms. Recent studies have further demonstrated that SIRT1 overexpression leads to increased expression of the efflux transporters MDR1 and MRP1 in drug-resistant cells and in tumor biopsies from cancer patients, thereby facilitating the removal of daunorubicin (DNR) from AML cells and contributing to treatment failure [9,26,29]. Interestingly, our results demonstrate that the expression levels of both SIRT1 and MDR1 are significantly reduced in chemoresistant cells treated with SC, either alone or in combination with Ara-C or DNR. These findings suggest that SC, when used alongside antileukemic agents, could inhibit the efflux of DNR in resistant cells, thereby resensitizing them to the drug. This is consistent with previous findings by Oh et al. [53], who reported that Murensin G, a natural compound, downregulates SIRT1 overexpression and MDR1 levels, resulting in the re-sensitization of cancer cells to chemotherapeutic agents. Additionally, we observed that SC, whether administered alone or with Ara-C, reduces the nuclear translocation of SIRT1, indicating that SC is likely the primary modulator of SIRT1 expression and activity. Notably, cytarabine-resistant cells have been shown to overexpress SIRT1 [9,26,29], and treatment with the Ara-C/SC combination appears to counteract this effect. While Ara-C alone may promote the nuclear localization of SIRT1, contributing to chemoresistance, the presence of SC significantly reduces both total and nuclear SIRT1 levels. This effect may underlie the observed reversal of chemoresistance, as it correlates with decreased proliferation and increased apoptosis in resistant cells. Importantly, the nuclear downregulation of SIRT1 could restore the activity of tumor suppressor genes such as p53, Ku70, FoxO1, and elements of the JNK/c-Jun signaling pathway, thereby promoting pro-apoptotic and antiproliferative responses [9,26]. These observations support the hypothesis that targeting SIRT1, directly or indirectly through agents like SC, could be a promising strategy to overcome drug resistance in AML. However, further investigations are necessary to delineate the precise molecular mechanisms by which SC regulates SIRT1 localization and function and to confirm whether these effects are reproducible in primary AML samples and in vivo models.

Although SIRT1 overexpression in cytarabine-resistant cells [26] does not directly regulate the expression of ENT1 or dCK, our results demonstrate that SC, in combination with antineoplastic agents, significantly reverses Ara-C resistance by upregulating ENT1 and dCK expression. These findings align with those reported by Takagaki et al. [54], who showed that increasing ENT1 expression restores Ara-C sensitivity in chemoresistant cells harboring wild-type genetic features. Moreover, it is well established that SIRT1 overexpression activates the JNK/c-Jun pathway [31], which negatively regulates ENT1 expression through c-Jun-mediated transcriptional repression [55]. Based on our data, we hypothesize that SIRT1 downregulation could attenuate c-Jun-dependent transcriptional repression, thereby permitting ENT1 overexpression and enhancing Ara-C uptake in resistant cells. Although further studies are required to confirm this regulatory mechanism, our findings strongly suggest that the combined treatment with SC and antileukemic drugs exerts a dual effect: (1) upregulation of ENT1 and dCK, facilitating Ara-C uptake and intracellular activation [3,16]; and (2) downregulation of SIRT1 and MDR1, preventing DNR efflux. This coordinated modulation ultimately leads to reduced cell proliferation, increased apoptosis, and improved survival in tumor-bearing mice when compared to monotherapy regimens.

## 4. Materials and Methods

### 4.1. Therapeutic Agents

Sodium caseinate was obtained from the Spectrum company (New Brunswick, NJ, USA). Cytarabine and daunorubicin were purchased from the Pfizer company (New York, NJ, USA). Stock solutions prepared in PBS were stored at −20 °C.

### 4.2. Cell Culture

WEHI-3 mouse cells were obtained from the American Type Culture Collection (Manassas, VA, USA), whereas cytarabine-resistant WEHI-CR50 cells were obtained in the Hematopoiesis and Leukemia Laboratory (Mexico) [16]. Both cell lines were cultured in Iscove’s Modified Dulbecco’s Medium (IMDM) (Gibco BRL, Grand Island, NY, USA), supplemented with 10% fetal bovine serum (FBS) (Gibco BRL, USA), 1.1 µM of beta-mercaptoethanol (Gibco BRL, USA), 100 U/mL penicillin, and 100 mg/mL streptomycin (Gibco, BRL, USA). The cells were maintained at 37 °C and under 5% CO_2_ with reseeding every 48 h.

### 4.3. Proliferation Assay

For cell proliferation assays, cells were seeded in quadruplicate in 96-well plates (2000 cells/well) and incubated with increasing concentrations of Ara-C (0, 1233.7, 1850.5, 3084.2, 4317.9, 5551.6, and 6785.3 nM), SC (0, 2, 4, and 8 mg), or DNR (0, 5, 10, 20, 40, 80, and 160 nM). Cells were stimulated with PBS as the control group. The obtained data were used to determine the 25% and 50% inhibitory concentrations (IC_25_ and IC_50_) for each treatment using a crystal violet assay.

To evaluate the effect of combined therapies on cell proliferation, chemoresistant WEHI-CR50 cells were exposed to different combinations with IC_25_ and IC_50_ concentrations of SC, Ara-C, or DNR. After 72 h of culture, viable cells were evaluated by a crystal violet assay as previously described [16]. The crystals were dissolved with acetic acid, and the absorbance of the reaction was determined to 570 nm using an automated microplate reader (Bio-Rad, Hercules, CA, USA).

### 4.4. Apoptosis Assay

WEHI-CR50 cells were seeded in 60 mm cell culture dishes at a density of 500,000 cells/dish. After 48 h of incubation in the absence or presence of SC, Ara-C, or DNR alone or in combination, cells were harvested, washed with PBS, suspended in 1× binding buffer at 1 × 10^5^ cells/mL, and then stained with annexin V-PE/7-AAD (Becton Dickinson, Franklin Lakes, NJ, USA) according to the manufacturer’s instructions. The fluorescence of annexin V-PE/7-AAD (at 528 nm and 650 nm, respectively) was analyzed using a BD FACSAria II flow cytometer (BD Biosciences, San Jose, CA, USA).

### 4.5. Real-Time RT-PCR

To evaluate the expression levels of genes associated with sensitivity to cytarabine (*ent1* and *dck*), multidrug resistance (*mdr1* and *mrp1*), and gene tumor promoter (*sirt1*), we performed quantitative real-time reverse transcription PCR. Briefly, WEHI-CR50 cells exposed to the different treatments for 72 h were used for RNA extraction using the Trizol reagent (Invitrogen, Waltham, MA, USA), according to the manufacturer’s instructions. Total RNA was quantified, and its integrity was determined on a 1% agarose gel. Real-time RT-PCR was performed using the SYBR Green system in an Applied Biosystems USA thermocycler with the following conditions: forty amplification cycles of 95 °C for 10 s; 60 °C for 30 s; an 72 °C for 15 s. Primer sequences were as follows: *ent1*, forward, 5′-CTGGAAAGGCGTAGAGGCTG-3; reverse, 5′-CTTCCCTTCGCAGACTGCTT-3′; *dck,* forward, 5′-AGCAGTGAGTCTGGAGGTAG-3; reverse, 5′-GAGAAGGCAGAGAAGGCTGG-3′; *mdr1,* forward, 5′-GTGGTGTCATTGTGGAGCAAG-3; reverse, 5′-GCATCAGTGTCACTCTGGGATC-3′; *mrp1,* forward, 5′-CAGTGGTTCAGGGAAGGATTTA-3; reverse, 5′-CACTGTGGGAAGACGAGTTGCT-3′; *sirt1,* forward, 5′-CGGCTACCGAGGTCCATATAC-3; reverse, 5′-CAGCTCAGGTGGAGGAATTGT-3′; *β-actin,* forward, 5′-CACTGTCGAGTCGCGTCC-3; reverse, 5′-CGCAGCGATATCGTCATCCA-3′. The fold change in the relative mRNA expression of each studied gene was calculated and normalized to the expression of the housekeeping gene β-actin using the double delta CT (2^−ΔΔCT^) method.

### 4.6. Immunofluorescence

The WEHI-CR50 cells were fixed with 4% paraformaldehyde (PFA), permeabilized with 0.25% Triton X-100 for 15 min, washed twice with PBS, and blocked with IgG-free-albumin 1% bovine serum albumin (Sigma-Aldrich, St. Louis, MO, USA) for 1 h at room temperature. The cells were incubated overnight at 4 °C with the primary antibody: Alexa Fluor^®^ 488 mouse anti-SIRT1 antibody (1:100 dilution; catalog 19A7AB4, Abcam, Cambridge, UK), Alexa Fluor^®^ 594 mouse anti-ENT1 antibody (1:200 dilution; catalog sc-377283, Santa Cruz Biotechnology, Dallas, TX, USA), Alexa Fluor^®^ 488 mouse anti-dCK antibody (1:200 dilution; catalog sc-393098, Santa Cruz Biotechnology), and Alexa Fluor^®^ 546 mouse anti-MDR1 antibody (1:100 dilution; catalog sc-55510, Santa Cruz Biotechnology) were used for immunofluorescence staining. The next day, cells were rinsed three times with PBS and counterstained with 6-diamidino-2-phenylindole (Vector Laboratories, Newark, CA, USA) to reveal the nuclei. Immunofluorescence experiments were performed at least three times in the different groups and evaluated using a confocal inverted microscope (TCS-SP2, Leica, Heidelberg, Germany). SIRT1 nuclear translocation was quantified as the percentage of the SIRT1 nuclei-positively stained cells of the total cells that were counted from three randomly chosen fields from three different experiments per treatment. Fifty nuclei were counted for each experimental condition.

### 4.7. Kaplan–Meier Survival Curves

All in vivo experiments were conducted in accordance with Mexican legislation NOM-062-ZOO-1999 (SAGARPA, Mexico City, Mexico) and the Guide for the Care and Use of Laboratory Animals of the U.S. National Institutes of Health (NIH). Experimental procedures were approved by the Bioethics and Safety Committee of the Faculty of Higher Studies Zaragoza (FESZ) under protocol number FESZ/DEPI/CI/216/14, ensuring compliance with both national and international standards for animal welfare. We followed a previously described implantation and treatment protocol, in which SC at a dose of 2 mg/kg demonstrated efficacy in both parental and chemoresistant WEHI-3 leukemia models [17].

A total of 40 healthy male mice (4 months old) were randomly divided into eight groups, each with five mice. Group I was set as the healthy control. Groups II to IX were mice inoculated intraperitoneally with 2.5 × 10^5^ WEHI-CR50 cells. A total of 24 h post-inoculation, treatments were initiated and administered intraperitoneally every 48 h for a total of 24 doses per animal. Group II consisted of leukemic mice (untreated), while group III, IV, V, and VI received PBS (vehicle), SC (2 g/kg), DNR (0.5 mg/kg), and Ara-C (3 mg/kg), respectively. Groups VII and VIII were treated with the SC + Ara-C or SC + DNR combinations, respectively. Mice were kept in groups of five per cage under aseptic conditions, with ad libitum access to food and water, and were maintained in a climate-controlled environment with a 12 h light/dark cycle. Animals were monitored daily for survival, and the time of death (in days) was recorded as the experimental endpoint for each mouse.

### 4.8. Statistical Analysis

All individual experiments were performed in triplicate or quadruplicate. Differences between means from the two different groups were analyzed using Student’s t-test. One-way or two-way ANOVA, followed by Tukey’s test, was used to analyze significant differences between the different experimental groups. Statistical analysis of the Kaplan–Meier survival curves was performed using the log-rank test followed by the Holm–Sidak method for pairwise multiple comparison tests. All values in graphs represent the mean ± standard deviation (SD). Statistical significance is indicated as *p* < 0.05, *p* < 0.01, *p* < 0.001, and *p* < 0.0001. Tests were performed using the GraphPad Prism 8 software.

## 5. Conclusions

The therapeutic strategy of employing natural products with chemotherapeutic agents shows that SC is a potent antileukemic agent capable of reducing the proliferation of chemoresistant cells. This beneficial effect occurs through the following: (i) Upregulating the expression of ENT1 and dCK, key molecules involved in the cellular uptake and metabolism of Ara-C, (ii) downregulating genes associated with chemoresistance, such as SIRT1 and MDR1. As a result, apoptosis is enhanced, and the survival of tumor-bearing mice injected with chemoresistant cells is improved. (iii) This combined therapy not only reduces drug resistance but also minimizes the concentration of chemotherapeutic agent compared to monotherapy, indicating that these components resensitize cytarabine-resistant AML cells.

## Data Availability

The datasets generated and/or analyzed during the present study are available from the corresponding author upon reasonable request.

## Abbreviations

Cytarabine (Ara-C), acute myeloid leukemia (AML), sodium caseinate (SC), daunorubicin (DNR), quantitative reverse transcriptase–polymerase chain reaction (qRT-PCR), equilibrative nucleoside transporter 1 (ENT1), deoxycytidine kinase (dCK), multidrug-resistance-associated transporters (MDR1), silent information regulator 1 homolog 1 (SIRT1), bone marrow mononuclear cells (BMMNC), Iscove’s Modified Dulbecco’s Medium (IMDM), inhibitory concentrations (IC), phosphate-buffered saline (PBS), ribonucleic acid (RNA), paraformaldehyde (PFA), National Institutes of Health (NIH), intraperitoneally (I.P.), standard deviation (SD).
